# Bilateral Symmetrical Herpes Zoster in an Immunocompetent 15-Year-Old Adolescent Boy

**DOI:** 10.1155/2015/121549

**Published:** 2015-01-27

**Authors:** Alexander K. C. Leung, Benjamin Barankin

**Affiliations:** ^1^The Alberta Children's Hospital, The University of Calgary, Calgary, AB, Canada T2M 0H5; ^2^Toronto Dermatology Centre, Toronto, ON, Canada M3H 5Y8

## Abstract

Herpes zoster is uncommon in immunocompetent children. The bilateral symmetrical occurrence of herpes zoster lesions is extremely rare. We report a 15-year-old immunocompetent Chinese adolescent boy who developed bilateral symmetrical herpes zoster lesions. To our knowledge, the occurrence of bilateral symmetrical herpes zoster lesions in an immunocompetent individual has not been reported in the pediatric literature.

## 1. Introduction

Herpes zoster, also known as shingles, is caused by reactivation of endogenous latent varicella-zoster virus (VZV) that resides in a sensory dorsal root ganglion [1]. Herpes zoster can develop any time after a primary infection with VZV (i.e., varicella or chickenpox) or varicella vaccination [1]. The activated virus travels back down the corresponding cutaneous nerve to the adjacent skin, causing typically a painful, unilateral vesicular eruption in a restricted dermatomal distribution. Herpes zoster is more common in persons with relative cell-mediated immunologic compromise such as elderly individuals or patients with an immunosuppressive illness or receiving immunosuppressive therapy. Immunocompromised individuals have a 20 to 100 times greater risk than immunocompetent individuals of the same age [2]. The bilateral symmetrical occurrence of herpes zoster lesions is extremely rare especially in immunocompetent children. We report a case of a 15-year-old immunocompetent Chinese adolescent boy with bilateral symmetrical herpes zoster lesions along T7, T8, and T9 dermatomes. To our knowledge, the occurrence of bilateral symmetrical herpes zoster lesions in an immunocompetent individual has not been reported in the pediatric literature.

## 2. Case Report

A 15-year-old Chinese boy presented with a bilateral and symmetrical painful eruption on the upper abdomen of 7 days' duration. The eruption was preceded by a 2-day history of malaise and low grade fever. He did not have the varicella vaccine but had chickenpox at 3 years of age. His past health was otherwise unremarkable. In particular, he did not have recurrent or chronic infections. The patient did not have recent weight loss and was not on any medications. There was no history of recent travel. He did not have exposure to venereal or other infectious diseases. The family history was noncontributory.

**Figure 1:**
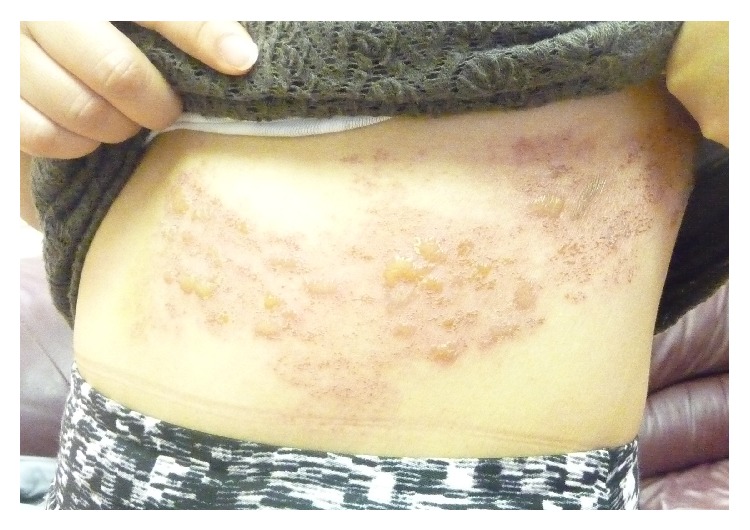
Bilateral, symmetrically distributed herpes zoster lesions along T7, T8, and T9 dermatomes.

Physical examination revealed multiple vesicles/bullae on an erythematous base, distributed bilaterally and symmetrically in a band-like distribution along T7, T8, and T9 dermatomes (Figure 1). The rest of the physical examination was unremarkable. His weight was 76 kg (90th percentile) and height 178 cm (70th percentile). His vital signs were normal and he was not in distress. There was no lymphadenopathy in the axillary or groin area, no organomegaly, and no muscle wasting.

Laboratory investigations revealed hemoglobin of 12.8 g/dL and white blood cell count of 7.8 × 10^9^/L with a normal differential count. His immunoglobulin levels were normal. The patient was treated with acyclovir 800 mg five times a day for 7 days. The blistering and discomfort resolved in 14 days, and the secondary dyspigmentation took 3 months to completely fade.

## 3. Discussion

In herpes zoster, the onset of disease is usually heralded by pain within the dermatome and precedes the lesions by 48 to 72 hours. An area of erythema then follows and precedes the development of a group of vesicles in the distribution of the dermatome that corresponds to the infected dorsal root ganglion. The diagnosis of herpes zoster is mainly made clinically, based on the distinctive clinical appearance and symptomatology. Laboratory tests usually are not necessary unless the rash is atypical.

In herpes zoster, usually one or, less commonly, two or three adjacent dermatomes are affected. The lesions typically do not cross the midline [1]. In individuals with immunodeficiency, the lesions may involve multiple contiguous, noncontiguous, bilateral, or unusual dermatomes. Dissemination occurs in 2 to 10% of immunocompromised individuals but rarely in immunocompetent individuals [1, 3]. Our patient was immunocompetent based on the history (unremarkable past health, absence of recurrent infections, or weight loss), physical findings (no muscle wasting, absence of fever, lymphadenopathy, or organomegaly), laboratory tests (normal complete blood count and immunoglobulin levels), and excellent response to oral acyclovir with complete recovery. The patient was not tested for HIV because he did not have venereal exposure, and there was no sign suggestive of HIV or immunodeficiency.

The simultaneous occurrence of herpes zoster in two noncontiguous dermatomes involving different halves of the body, also termed herpes zoster duplex bilateralis, is distinct from disseminated VZV infection. Herpes zoster duplex bilateralis is rarely reported, especially in immunocompetent individuals [4–7]. It is estimated that herpes zoster duplex bilateralis accounts for less than 0.1% of all herpes zoster cases and occurs mainly in immunocompromised individuals [7].

The bilateral symmetrical occurrence of herpes zoster lesions, also known as herpes zoster duplex symmetricus, is extremely rare, especially in immunocompetent individuals [8]. Presumably, such occurrence is related to a high VZV genome load in the dorsal root ganglia in the same dermatome bilaterally. A perusal of the literature revealed only 3 cases of herpes zoster occurring bilaterally in the same dermatome [8–10]. In 1947, Thomas reported a 33-year-old woman who had herpes zoster in the upper sacral areas, hips, and upper part of the buttocks bilaterally [9]. In 2003, Arfan-ul-Bari et al. described a 24-year-old otherwise healthy man who had herpes zoster lesions over the lower chest in a horizontal band-like distribution bilaterally [10]. In 2006, Brandon et al. reported a 39-year-old female who developed bilateral herpes zoster at the T8 dermatome level on the fourth day after bilateral thoracoscopic splanchnicectomy for chronic severe visceral pain [8]. Her past health included 6 years of chronic pancreatitis secondary to hyperlipidemia and associated insulin-dependent diabetes mellitus. To our knowledge, our patient represents the first immunocompetent patient who had bilateral symmetrical herpes zoster in the pediatric age group.

It is known that vaccine-associated herpes zoster is milder than herpes zoster after wild-type varicella [1]. As such, there is a need for prevention of VZV infection through universal childhood immunization [11].

## 4. Conclusion

The bilateral symmetrical occurrence of herpes zoster lesions is extremely rare, especially in immunocompetent individuals. We report a Chinese immunocompetent teenager who had bilateral symmetrical herpes zoster lesions. To our knowledge, the occurrence of bilateral symmetrical herpes zoster lesions in an immunocompetent individual has not been reported in the pediatric literature.
